# Negative energy dust acoustic waves evolution in a dense magnetized quantum Thomas–Fermi plasma

**DOI:** 10.1038/s41598-022-20174-y

**Published:** 2022-09-23

**Authors:** M. Abd-Elzaher, A. Atteya

**Affiliations:** 1 Department of Basic and Applied Sciences, Faculty of Engineering, Arab Academy for Science, Technology and Maritime, Transport, Alexandria, Egypt; 2 Department of Physics, Faculty of Science, Alexandria University, P.O. 21511, Alexandria, Egypt

**Keywords:** Plasma physics, Space physics

## Abstract

Propagation of nonlinear waves in the magnetized quantum Thomas–Fermi dense plasma is analyzed. The Zakharov–Kuznetsov–Burgers equation is derived by using the theory of reductive perturbation. The exact solution contains both solitary and shock terms. Also, it is shown that rarefactive waves propagate in most cases. Both the associated electric field and the wave energy have been derived. The effects of dust and electrons temperature, dust density, magnetic field magnitude, and direction besides the effect of the kinematic viscosity on the amplitude, width, and energy of the formed waves are discussed. It is shown that the negative energy wave is formed and its value is enhanced due to the increase of the kinematic viscosity and the ambient magnetic field which lead to an increase in the instability. The present results are helpful in controlling the stabilization of confined Thomas–Fermi dense magnetoplasma that are found in white dwarfs and in the high-intensity laser-solid matter interaction experiments.

## Introduction

The dusty plasma waves investigations occur when the dust-acoustic waves (DAWs) were theoretically predicted first by Rao et al. 1990^1^ and were later confirmed experimentally^2–6^. Since then numerous studies have been presented on different types of waves in the dusty plasma^5–7^. The linear and nonlinear properties of plasma were investigated experimentally and have been confirmed to depend on the plasma particles’ velocity distribution functions. Moreover, the contamination due to charged micron or submicron-sized dust grains affects the properties of electron-ion plasma and new oscillation modes released and can be studied in plasmas, e.g., DAWs^1^, dust ion-acoustic (DIA) mode^8^.

The electron density in fluid hydrodynamic equations was considered as Boltzmann distribution in most of the works that were used to investigate DAWs. However, in white dwarfs as an example of astrophysical environments, the electron and ion density numbers are about $10^{30}\,{\rm cm}^{-3}$ and exhibit relatively weak interactions. In this case, we can use the Thomas–Fermi approximation to describe inertialess degenerate electrons^9–11^. The dust is taken as classical and dynamic, while electrons and ions are taken to be Thomas–Fermi density distribution. The Thomas–Fermi distribution for electrons is employed by Dubinov and Dubinova^11^. They investigated the subsonic periodic and supersonic solitary waves occurrence. The cylindrical and spherical KdV equation was derived for nonplanar solitary waves^12^. The unmagnetized Thomas–Fermi electron-positron-ion plasma was also considered to study the solitary waves^13^. Sabry et al.^14^ studied the obliquely explosive propagating solitary waves in dense magnetoplasma with Thomas–Fermi degenerate electrons. The solitary waves and double layer properties of dusty magnetoplasma have been also investigated^15,16^. Later, derivation of the Zakharov–Kuznetsov (ZK) and Zakharov–Kuznetsov–Burger (ZKB) equations found that the DA shock and solitary waves are affected by the variation of the concentration, viscosity, and temperature of the dust^17^. The propagations of solitary and rogue waves are investigated in a degenerate Thomas–Fermi thermal dusty plasma through the transverse effects of velocity perturbation^18^. Hafez et al. also investigated ion-acoustic waves in an unmagnetized Thomas–Fermi plasma for both non-relativistic and ultra-relativistic degenerate electrons systems by the derivation of KdV equation and using the Riccati equation mapping method to solve it^19^. Obliquely propagating waves in a dense degenerate cold Thomas–Fermi magnetoplasma were investigated by Irfan et al.^20^. They employed the Sagdeev pseudopotential theory to derive an energy-balance equation. It was demonstrated that the wave characteristics depend upon the system parameters. The degenerate electrons in dense magnetoplasma were taken into consideration in the study of overtaking collision of unidirectional DAWs in the Thomas–Fermi dense magnetoplasma^21^.

The negative energy wave emerges when a reduction of the total energy of the system accompanied the wave excitation. The reduction or removal of the energy from the wave can be achieved by dissipation or coupling to another wave of positive energy results in the instability of the system due to the wave growth^22^. The reduction in the wave energy occurs when the energy becomes negative and signifies an increase in both the wave amplitude and, the energy absolute value. These negative energy waves are adequate to occur only in nonequilibrium systems and, in systems containing neutral fluid shear flows or charged-particle beams.

The negative energy waves were introduced by Cairns to describe the stability of fluid flows^23^. Two instability forms, reactive and dissipative were illustrated, which leads to the emergence of the negative energy wave^24^. Also, negative energy waves were taken in the resistive wall mode, which is magnetically confined plasma instability^25^. It is different from the resistive wall amplifier, but a subtle link between them in the presence of plasma flow^26^.

Ryutova shows that negative energy wave is important for the energy transfer to the higher solar atmosphere^27^. The boundary of discontinuous shear flow in an incompressible plasma system was investigated by Ruderman and Goossens^28^ . The Alfvén surface wave propagation and the solution for its negative energy were derived by considering constant flow on one side and viscosity on the other side. They obtained two copropagating modes of phase speed, with the fact that the negative energy was associated with the slower one. Also, they found that the increase in viscosity coefficient leads to an increase in the growth rate of the instability. Ruderman found that the standing surface wave growth rate equals the difference between the propagating backward negative energy wave and the propagating forward positive energy wave, this means that the negative energy wave exceeds the positive energy wave by the growth rate^29^. The appearance of negative energy surface waves in an incompressible cylindrical Plasma was investigated by Yu and Nakariakov^30^. They found that the instability depends strongly on the shear flow speed and on the plasma temperature.

However, to the best of the authors’ knowledge, no attempt has been made considering the negative energy waves propagation in the magnetized quantum Thomas–Fermi plasma. Therefore, in this work, we derive the ZKB equation to study the negative energy waves associated with the DAWs. This manuscript is organized as follows. The governing equations and the derivation of the magnetized ZKB equation is in Derivation of the ZKB equation section. The wave solution, the associated electric field, and the wave energy are in The ZKB equation solution section. The numerical investigations and discussions are provided in Numerical investigations and discussion section. Finally, Conclusions section is devoted to the conclusions.

## Derivation of the ZKB equation

We consider magnetized quantum Thomas–Fermi dense plasma consisting of negatively charged dust particles with degenerate electrons and ions obeying the Fermi-Dirac distributions. The external magnetic field $\mathbf{B}_{0}$ has confined the plasma system and it is along the z-direction, i.e., $\mathbf{B}_{0}=\hat{z}B_{0}$ where $\hat{z}$ is the unit vector along the z-axis and $B_{0}$ is the strength of the magnetic field. The quasineutrality condition is $N_{e0}=N_{i0}-N_{d0}Z_{d0}$ at equilibrium, where $N_{s0}$ is the sth species equilibrium density($s=e$, *i*, and *d* for electrons, ions, and negatively charged dust grains, respectively), $Z_{d0}$ is the equilibrium state dust charge. The dynamics of the DA for the Thomas–Fermi magnetoplasma are governed by^21^

1 $$\begin{aligned} \left. \begin{array}{c} \frac{\partial N_{d}}{\partial t}+\nabla .(N_{d}\mathbf{U}_{d})=0,\\ \frac{\partial \mathbf{U}_{d}}{\partial t}+\mathbf{U}_{d}.\nabla \mathbf{U} _{d}=\nabla \psi -\Omega \mathbf{U}_{d}\times \hat{z}-\sigma _{d}N_{d}\nabla N_{d}+\eta \nabla ^{2}\mathbf{U}_{d},\\ \nabla ^{2}\psi =\mu _{e}N_{e}-\mu _{i}N_{i}+N_{d},\\ N_{e}=\left( 1+\sigma _{i}\psi \right) ^{3/2},\\ N_{i}=\left( 1-\psi \right) ^{3/2}, \end{array} \right\} \end{aligned}$$

where $N_{s}$ is the normalized number density, $U_{d}$ dust fluid velocity that normalized by the DA speed $C_{d}=\left( 2Z_{d0}k_{B}T_{Fi} /m_{d}\right) ^{1/2},$ $\psi$ is the wave potential that normalized by $2k_{B}T_{Fi}/e$. $\Omega =\omega _{cd}/\omega _{pd}$ is the normalized dust gyro-frequency with $\omega _{cd}=eZ_{d0}B/m_{d}$ and $\omega _{pd}=\left( 4\pi Z_{d0}^{2}n_{d0}e^{2}/m_{d}\right) ^{1/2}$. Also, $\sigma _{d}=T_{d}/T_{Fi}Z_{d0}$, $\mu _{i}=n_{i0}/Z_{d}n_{d0}$, and $\mu _{e}=n_{e0}/Z_{d}n_{d0},$ are the dust temperature-to-ion Fermi temperature ratio, the ion concentration, and electron concentration, divided by $n_{d0}Z_{d0}$, respectively, with *e* is the electronic charge, $k_{B}$ is the Boltzmann constant. $\sigma _{i}=T_{Fi}/T_{Fe}$ is the ion-to-electron Fermi temperature ratio. The charge-neutrality condition at equilibriumbecomes $\mu _{i=}\mu _{e+1}$. The space variable is normalized by $\lambda _{0}=\left( 2k_{B}T_{Fi}/4\pi Z_{d}n_{d0}e^{2}\right) ^{1/2},$ and the time variable *t* is normalized by $\omega _{pd}^{-1}$.

To derive the ZKB equation, the stretching of the independent variables x, y, and t is defined as^7^

2 $$\begin{aligned} X=\epsilon ^{1/2}x,Y=\epsilon ^{1/2}y,Z=\epsilon ^{1/2}(z-v_{0}t),T=\epsilon ^{3/2}t,\eta =\epsilon ^{1/2}\eta _{0}, \end{aligned}$$

where $\epsilon$ is a formal small expansion parameter measuring the strength of the system nonlinearity, $v_{0}$ is the phase velocity. The dependent variables can be expanded in power series of $\epsilon$ as follows:

3 $$\begin{aligned} \left. \begin{array}{c} N_{d}=1+\epsilon N_{d}^{(1)}+\epsilon ^{2}N_{d}^{(2)}+\epsilon ^{3}N_{d} ^{(3)}+...,\\ U_{dx,y}=\epsilon ^{\frac{3}{2}}U_{dx,y}^{(1)}+\epsilon ^{2}U_{dx,y} ^{(2)}+\epsilon ^{\frac{5}{2}}U_{dx,y}^{(3)}+...,\\ U_{dz}=\epsilon U_{dz}^{(1)}+\epsilon ^{2}U_{dz}^{(2)}+\epsilon ^{3}U_{dz} ^{(3)}+...,\\ \psi =\epsilon \psi ^{(1)}+\epsilon ^{2}\psi ^{(2)}+\epsilon ^{3}\psi ^{(3)}+..... \end{array} \right\} \end{aligned}$$

Substituting Eqs. (2) and (3) into Eqs. (1) and the same powers of $\epsilon$ are collected, which gives for the lowest orders perturbed quantities;

4 $$\begin{aligned} N_{d}^{(1)}=\frac{-\psi ^{(1)}}{v_{0}^{2}-\sigma _{d}},U_{dz}^{(1)}=\frac{v_{0}\psi ^{(1)}}{v_{0}^{2}-\sigma _{d}}. \end{aligned}$$

The propagation phase speed of the DA waves in the magnetized dusty plasma is given by

5 $$\begin{aligned} v_{0}=\sqrt{\frac{2+3\mu _{i}\sigma _{d}+3\mu _{e}\sigma _{d}\sigma _{i}}{3\mu _{i}+3\mu _{e}\sigma _{i}}.} \end{aligned}$$

**Figure 1:**
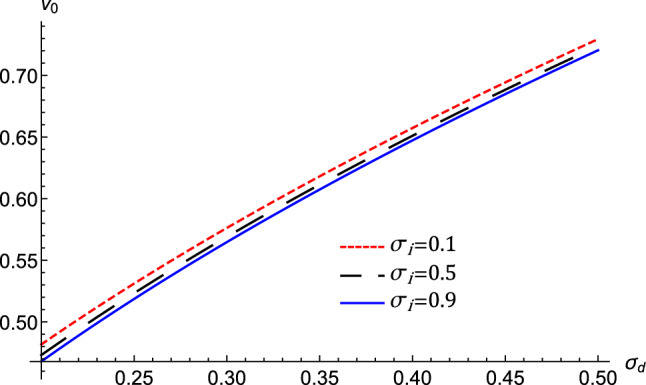
The variation of the phase speed $v_{0}$, represented by Eq. (5) against $\sigma _{d}$ for different values of $\sigma _{i}$ at $\mu _{e}=18$.

We investigate the influence of important plasma parameters on the phase speed because we are interested in analysing the characteristics of acoustic modes in the plasma system under consideration. The phase speed of the acoustic wave is dependent on many system parameters, as shown by Eq. (5). Accordingly, Fig. 1 illustrates the dependence of $v_{0}$ on the ion-to-electron Fermi temperatures ratio, $\sigma _{i}$, the dust temperature-to-ion Fermi temperature ratio, $\sigma _{d}$. Combining the next higher-orders contributions lead to

6 $$\begin{aligned} \left. \begin{array}{c} U_{dx}^{(1)}=\frac{-v_{0}^{2}}{\Omega \left( v_{0}^{2}-\sigma _{d}\right) }\frac{\partial \psi ^{(1)}}{\partial Y},\\ U_{dy}^{(1)}=\frac{v_{0}^{2}}{\Omega \left( v_{0}^{2}-\sigma _{d}\right) }\frac{\partial \psi ^{(1)}}{\partial X},\\ U_{dx}^{(2)}=\frac{-v_{0}^{3}}{\Omega \left( v_{0}^{2}-\sigma _{d}\right) }\frac{\partial ^{2}\psi ^{(1)}}{\partial X\partial Z},\\ U_{dy}^{(2)}=\frac{-v_{0}^{3}}{\Omega \left( v_{0}^{2}-\sigma _{d}\right) }\frac{\partial ^{2}\psi ^{(1)}}{\partial Y\partial Z}. \end{array} \right\} \end{aligned}$$

Now, the next higher order of $\epsilon$ gives rise to the following equation

7 $$\begin{aligned} \left. \begin{array}{c} \frac{\partial N_{d}^{(2)}}{\partial Z}=-\frac{2v_{0}}{\left( v_{0} ^{2}-\sigma _{d}\right) ^{2}}\frac{\partial \psi ^{(1)}}{\partial T} +\frac{\left( 3v_{0}^{2}+\sigma _{d}\right) }{\left( v_{0}^{2}-\sigma _{d}\right) ^{3}}\psi ^{(1)}\frac{\partial \psi ^{(1)}}{\partial Z}-\frac{1}{\left( v_{0}^{2}-\sigma _{d}\right) }\frac{\partial \psi ^{(2)}}{\partial Z}\\ -\frac{v_{0}^{4}}{\Omega ^{2}\left( v_{0}^{2}-\sigma _{d}\right) ^{2}} \frac{\partial ^{3}\psi ^{(1)}}{\partial X^{2}\partial Z}-\frac{v_{0}^{4} }{\Omega ^{2}\left( v_{0}^{2}-\sigma _{d}\right) ^{2}}\frac{\partial ^{3} \psi ^{(1)}}{\partial Y^{2}\partial Z}. \end{array} \right\} \end{aligned}$$

Substitute in the Poisson’s equation, we obtain a partial differential equation (PDE) in the form

8 $$\begin{aligned} \frac{\partial \psi ^{(1)}}{\partial T}+A\psi ^{(1)}\frac{\partial \psi ^{(1)} }{\partial Z}+B\frac{\partial ^{3}\psi ^{(1)}}{\partial Z^{3}}+C\left( \frac{\partial ^{3}\psi ^{(1)}}{\partial X^{2}\partial Z}+\frac{\partial ^{3} \psi ^{(1)}}{\partial Y^{2}\partial Z}\right) +D\left( \frac{\partial ^{2} \psi ^{(1)}}{\partial X^{2}}+\frac{\partial ^{2}\psi ^{(1)}}{\partial Y^{2} }+\frac{\partial ^{2}\psi ^{(1)}}{\partial Z^{2}}\right) =0. \end{aligned}$$

This PDE is recognized as the ZKB equation. The nonlinearity coefficient *A*, the dispersive, *B*, and the transverse, *C* terms are given by the expressions

9 $$\begin{aligned} \left. \begin{array}{c} A=\left( \frac{3v_{0}^{2}+\sigma _{d}-\frac{3}{4}\left( v_{0}^{2}-\sigma _{d}\right) ^{3}\left( \mu _{i}-\mu _{e}\sigma _{i}^{2}\right) }{2v_{0} \sigma _{d}-2v_{0}^{3}}\right) ,\\ B=\frac{\left( v_{0}^{2}-\sigma _{d}\right) ^{2}}{2v_{0}},\\ C=\frac{1}{2v_{0}}\left( \left( v_{0}^{2}-\sigma _{d}\right) ^{2} +\frac{v_{0}^{4}}{\Omega ^{2}}\right) ,\\ D=-\frac{\eta _{0}}{2}. \end{array} \right\} \end{aligned}$$

**Figure 2:**
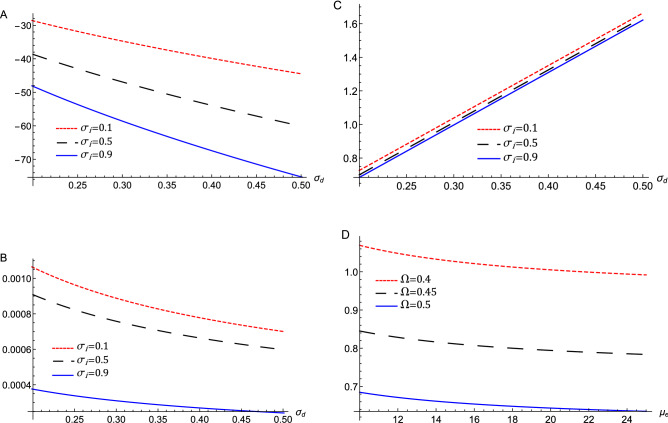
The variation of (**a**) the nonlinear term *A*, (**b**) the dispersive term *B*, (**c**) the transverse term *C* against $\sigma _{d}$ for different values of $\sigma _{i}$, at $\mu _{e}=18$, and (**d**) the transverse term *C* against $\mu _{e}$ for different values of $\ \Omega$ at $\sigma _{i}=0.5$, and $\sigma _{d}=0.3$ that are represented by Eq. (9).

The effects of $\sigma _{i}$, and $\sigma _{d}$ on these *A*, *B*, and, *C* coefficients are illustrated in Fig. 2. This figure depicts that the nonlinear coefficient is negative and its absolute value increases as $\sigma _{i}$, and $\sigma _{d}$ increase. Both *B* and *C* decrease as $\sigma _{i}$ increase while *B*(*C*) decreases (increases) as $\sigma _{d}$ increases. Figure 2d show that the transverse term, *C*, decreases as $\mu _{e}$, and the magnetic field through $\Omega$ increases, which mathematically related to the inverse proportionality relation between *C* and $\Omega$ as mentioned in Eq. (9).

## The ZKB equation solution

To transform the planar partial ZKB equations (8) into ordinary differential equations, we introduce the variable $\chi =lX+mY+nZ-u_{o}T$ where $\chi$ is the transformed coordinate relative to a frame that moves with the velocity $u_{o}$. *l*, *m*, and *n* are direction cosines of the wave propagation vector *k* with respect to *X*;*Y*, and *Z* axes, respectively. $u_{o}$ is the velocity of the moving frame normalized by dust acoustic speed.

Considering this transformation, Eq. (8) takes the form of an ordinary differential equation given by

10 $$\begin{aligned} -u_{o}\frac{d\psi ^{(1)}}{d\chi }+nA\psi ^{(1)}\frac{\partial \psi ^{(1)}}{\partial \chi }+\left[ Bn^{3}+Cn\left( l^{2}+m^{2}\right) \right] \frac{d^{3}\psi ^{(1)}}{d\chi ^{3}}+D\frac{d^{2}\psi ^{(1)}}{d\chi ^{2}}=0. \end{aligned}$$

Now, the general exact solution of Eq. (10) comprising both the dispersion and dissipative terms can be obtained by employing the hyperbolic tangent (tanh) method^31^, which is a method for deriving the traveling wave solutions of distinct types of nonlinear evolution equations. Therefore, the solution can be derived to be^32^

11 $$\begin{aligned} \psi ^{(1)}\left( \chi \right) =\frac{3}{25}\frac{D^{2}}{An^{2}\left[ Bn^{2}+C\left( 1-n^{2}\right) \right] }\left[ \begin{array}{c} 2-2\tanh \left( \frac{D}{10n\left[ Bn^{2}+Cn\left( 1-n^{2}\right) \right] }\chi \right) \\ +{\text {sech}}^{2}\left( \frac{D}{10n\left[ Bn^{2}+C\left( 1-n^{2}\right) \right] }\chi \right) \end{array} \right] . \end{aligned}$$

This ZKB equation, Eq. (10), contains both dispersion and dissipative effects contribution from which affect the wave potential eventual shape. It is noticed that this solution has been obtained in the region of parameter values where the nonlinearity, dispersion, and dissipative coefficients in the ZKB equation (10) are affected by the parameters’ values. Therefore, the solution covers the range of plasma parameters. The associated electric field can be represented according to the relation ($E^{(1)}=-\nabla \psi ^{(1)}$)^33^ and it takes the form

12 $$\begin{aligned} E^{(1)}\left( \chi \right)&=\frac{3}{125}\frac{D^{3}}{An^{3}\left[ Bn^{2}+C\left( 1-n^{2}\right) \right] ^{2}}{\text {sech}}^{2}\left( \frac{D}{10n\left[ Bn^{2}+C\left( 1-n^{2}\right) \right] }\chi \right) \nonumber \\&.\left[ \tanh \left( \frac{D}{10n\left[ Bn^{2}+Cn\left( 1-n^{2}\right) \right] }\chi \right) +1\right] . \end{aligned}$$

In addition to the above, the energy is also an important feature, which can be calculated as reported by Ko and Kuehl^34,35^ as

13 $$\begin{aligned} E_{n}&= {\displaystyle \int \limits _{-\infty }^{\infty }} \frac{\psi ^{(1)^{2}}}{v_{0}^{2}}\left( \chi \right) d\chi ,\nonumber \\ E_{n}&=\frac{24D^{2}}{125A^{2}v_{0}^{2}n^{4}\left[ Bn^{2}+C\left( 1-n^{2}\right) \right] ^{2}}. \end{aligned}$$

Motivated by these theoretical works, according to the energy equation, Eq. (13), the role of viscosity, dust temperature, and magnetic field effects may significantly play on the energy carried by the formed DA waves.

## Numerical investigations and discussion

In this section, we present numerical investigations of equations (5, 9, 11–13 to evaluate the phase velocity, amplitude, and width of the nonlinear dust acoustic structures in a magnetized quantum Thomas–Fermi dense plasma. For the numerical results, environments like white dwarfs parameters are used. Particularly, we analyze the effect of the unperturbed density ratio of ions-to-dust, $\mu _{i}$, electron-to-dust, $\mu _{e}$, the ion-to-electron Fermi temperatures ratio, $\sigma _{i}$, the dust temperature-to-ion Fermi temperature ratio, $\sigma _{d}$, the direction cosines of the wave vector along the z-axis, viscosity and magnetic field on the energy of the nonlinear DAWs and the associated electric field. It is realized from Fig. 1 that $v_{0}$ increases as electron Fermi temperature and dust temperature increase, while $v_{0}$ decreases as the ion Fermi temperature increases. This can be physically attributed to the fact that the restoring force is provided by the inertialess electrons. Accordingly, the increase of electrons energies and the dusts thermal pressure lead to an increase in the electron Fermi temperature $T_{Fe}$, and dust temperature, $T_{d}$^36^, respectively. This results in the increase of the phase velocity.

The nonlinearity coefficient *A*, the dispersive, *B*, and the transverse, *C* terms are dependent on $$\sigma _{i}$$, and $$\sigma _{d}$$ as depicted in Fig. 2a–c. The transverse term *C* is the only factor that depends on the magnetic field as depicted in Fig. 2. In comparison with Fig. 1, only the transverse term, $$\mathbf{C}$$ takes the same behavior of the phase velocity against $$\sigma _{i} $$, and $$\sigma _{d}$$ as it is directly proportional to $$v_{0} $$. The behavior of $$\mathbf{A}$$ and $$\mathbf{B}$$ is altered by comparing with the phase velocity against $$\sigma _{i}$$, and $$\sigma _{d}$$ as shown in Fig. 2a,b as they depend on $$\sigma _{i}$$, and $$\sigma _{d}$$ in the opposite way.

**Figure 3:**
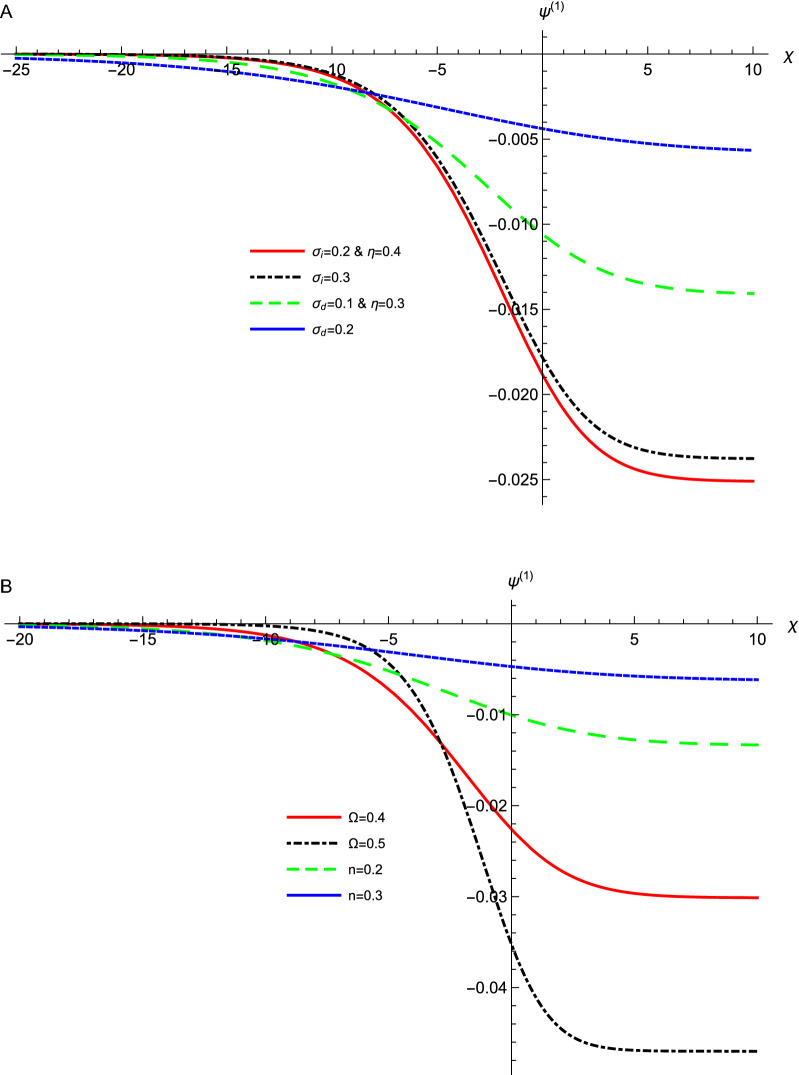
The evolution of the potential $\phi ^{(1)}$ of the DA waves that represented by Eq. (11) with $\chi$ at $\mu _{e}=18$, for different values of (**a**) $\sigma _{i}$, $\sigma _{d}$, and $\eta$ with $n=0.3$, and $\Omega =0.4$, (**b**) *n*, and $\Omega =$ with $\sigma _{i}=0.2$, $\sigma _{d}=0.1$, and $\eta =0.3$.

**Figure 4:**
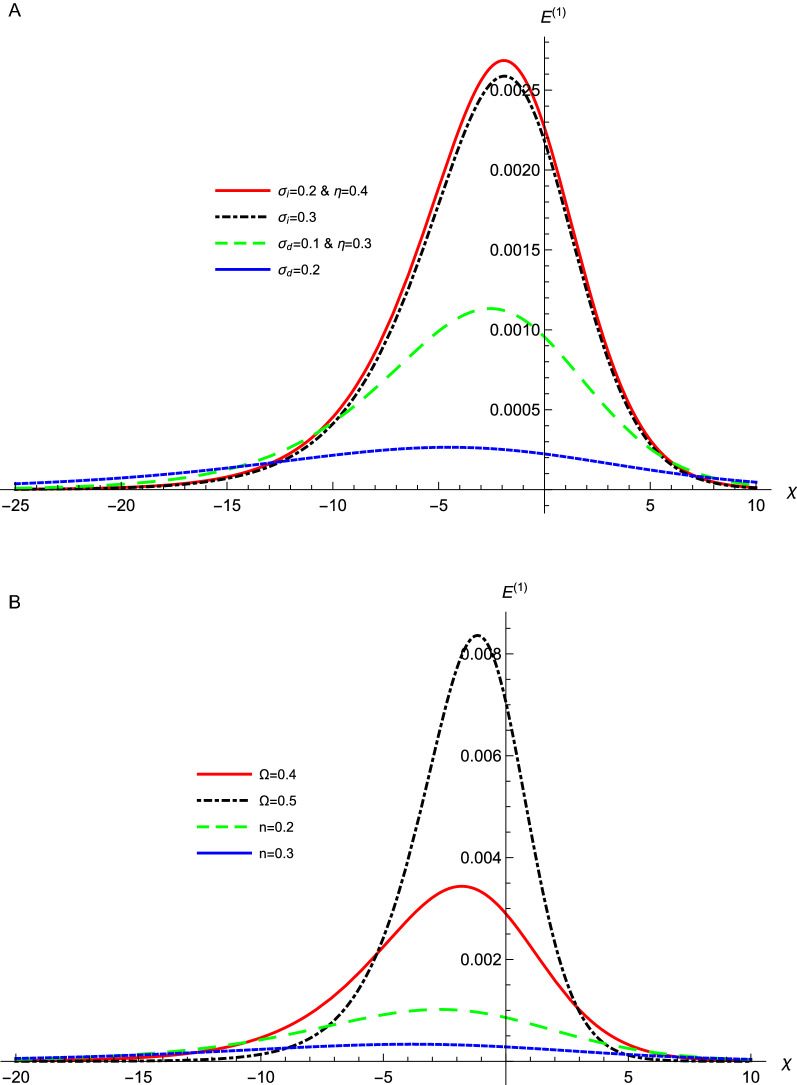
The evolution of the associated electric field, $E^{(1)}$of DA waves that represented by Eq. (12) with $\chi$ for the potentials those represented by Fig. 1, for different values of (**a**) $\sigma _{i}$, $\sigma _{d}$, and $\eta$ with $n=0.3$, and $\Omega =0.4$, (**b**) *n*, and $\Omega =$ with $\sigma _{i}=0.2$, $\sigma _{d}=0.1$, and $\eta =0.3$.

Figure 3 depicts the effect of various data values of $\sigma _{i}$, $\sigma _{d}$, $\eta$, *n*, and $\Omega$ on the profile of the DAWs of the ZKB equation, Eq. (11), against the coordinate $\chi$. It is clear that the dissipative term is dominant and a rarefactive shock profile is obtained. The amplitude of the formed shock waves decreases as $\sigma _{i}$, $\sigma _{d}$, and *n* increase. While, the amplitude increases as $\eta$, and $\Omega$ increase. All these parameters appear in *A*, *B*, *C*, and *D* where they affect the wave amplitude as presented in the $D^{2}/An^{2}\left[ Bn^{2}+C\left( 1-n^{2}\right) \right]$ term. It is concluded that the effect of increasing $\sigma _{i}$ and $\sigma _{d}$ on the nonlinear term is dominant in affecting the shock wave amplitude. Since *A* is inversely proportional to the amplitude and it inceases drastically in comparison with the behavior of *B* and *C* against $\sigma _{i}$ and $\sigma _{d}$. Accordingly, energetic electrons leads to larger phase velocity and larger amplitude shock waves, while inertial dusts with smaller temperature and smaller phase velocity results in larger amplitude shock waves. The width in our system, $10n\left[ Bn^{2}+C\left( 1-n^{2}\right) \right] /D)$, is affected by $\mathbf{B}$, $\mathbf{C}$, and $\mathbf{D}$ in different manners as they are multiplied by $\mathbf{n}$. According to Fig. 2, the dominant effect is attributed to the change in $\mathbf{D}$ since it emerges larger values against $\eta$ on comparing with those from $\mathbf{B}$ and $\mathbf{C}$. The associated electric field is depicted in Fig. 4. The electric field shows the same behavior where it increases (decreases) as $\eta$ and $\Omega$ ($\sigma _{i}$, $\sigma _{d}$ and *n*) increase. This means physically that, more energetic electrons (electrons with higher $T_{Fe}$ or lower $\sigma _{i}$) lead to an increase in the restoring force and in situ increase in the amplitude as shown in Figs. 3a and 4a. The dusts temperature $T_{d}$ increases as their thermal pressure increases, which leads the waves to be less negative that results in the decrease in the amplitude as depicted in Figs. 3a and 4a. The dissipation leads also to an increase in the amplitude as depicted also in these figures. Physically, this behavior is because the kinematic viscosity increase leads to an increase in dissipation and consequently causes strong shock waves and strong associated electric field structures. Also, one can predict that when the nonlinear DAWs approach the direction parallel to the magnetic field (*n* increases), the amplitude and the width of it shrink as shown in 3b and 4b, and from this point, we can predict physically that the DAWs are confined in the field direction. Also, the magnetic field increase leads to an increase in the force and in situ increase in the amplitude as depicted in the same figures. This results from the fact that the waves propagate across the magnetic field, where the compression of magnetic field lines provides the acoustic restoring force for the wave propagation^37^.

**Figure 5:**
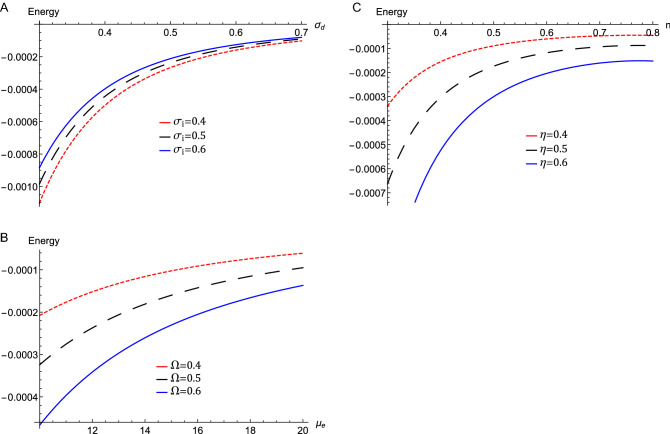
The evolution of the energy $E_{n}$ of the DA waves that represented by Eq. (13), (**a**) against $\sigma _{i}$ for different values of $$\sigma _{d}\ $$ with $\eta =0.8$, $n=0.4$, $\Omega =0.5$, and $\mu _{e}=12$, (**b**) against $\mu _{e}$ for different values of $$\Omega \ $$ with $\eta =0.8$, $n=0.4$, $\sigma _{i}=0.5$ and $\sigma _{d}=0.5$, (**c**) against *n* for different values of $$\eta \ $$ with $\sigma _{i}=0.5$, $\sigma _{d}=0.5$, $\Omega =0.5$, and $\mu _{e}=18$.

The variation of wave energy, $E_{n}$, versus $\sigma _{d}$ for different values of $\sigma _{i}$ is depicted in Fig. 5a. On the other hand, Fig. 5b shows the variation of $E_{n}$ versus $\mu _{e}$ for different values of $\Omega$. The variation of $E_{n}$ against *n* for different values of $\eta$ is depicted in Fig. 5c. We see in Figure 5 that the wave energy of obliquely propagating DAWs is higher for higher values of $\Omega$ and $\eta$. on the other side, the wave energy shrinks for higher values of *n*, $\mu _{e}$, $\sigma _{d}$, and $\sigma _{i}$. This can be inferred as previously mentioned due to the magnitude and the direction of the magnetic field, and dust inertia on the formed DAWs. Kinematic viscosity plays a key role in dissipation for the propagation of DA shocks. This coincides with the postulation that the growth of the wave by increasing the dissipation results in negative energy increase that is an indication of instability increase^38^. Also, the restoring force and inertia are affected by the electron and dust concentrations, respectively. Accordingly, $\mu _{e}$ affects significantly the energy of the DAWs.

## Conclusions

Based on the hydrodynamic model of magnetized quantum Thomas–Fermi dense plasma and rigorous development of nonlinear wave theory we have described DAWs evolution by the ZKB equation. The solution of this ZKB equation has been used to explore the development of the DAWs’ potential, electric field, and energy. The DAWs propagating in the background magnetic field direction are confined. While the field increase leads to larger energy. Our results coincide with those obtained by Infeld and Frycz^39^ that planar waves propagating parallel to the magnetic field become more unstable if the field is robust enough.

The dissipation involving plasma viscosity with wave dispersion and nonlinearity leads to nonlinear excitations in the form of shocks in plasmas. Also, we demonstrated that finite viscosity causes the formation of dissipative negative energy wave instability^30^. The increase of the electron density through $\mu _{e}$ leads to decelerating them^32^, as those obtained due to the increase of $\sigma _{i}$ and $\sigma _{d}$. Consequently, a decrease in the restoring force results in the reduction of wave energy. The theory was examined and the valid ranges were also investigated rigorously in the numerical simulations .The idea of negative energy waves is an essential scheme for classifying instabilities into dissipative and reactive^40^. Accordingly, This prooof of the existence of the negative energy waves helps control the stabilization of confined plasma^41^.

Our present results may be useful in understanding the nonlinear localized structures in the laboratory such as the high-intensity laser-solid matter interaction experiments and white dwarfs as an example of the space plasmas where Thomas–Fermi dense magnetoplasma occurs^42,43^.

## Data Availability

The data used to support the findings of this study are included within the article and available in ref.^21^
